# Ncm, a Photolabile Group for Preparation of Caged Molecules: Synthesis and Biological Application

**DOI:** 10.1371/journal.pone.0163937

**Published:** 2016-10-03

**Authors:** Sukumaran Muralidharan, Nathaniel D. A. Dirda, Elizabeth J. Katz, Cha-Min Tang, Sharba Bandyopadhyay, Patrick O. Kanold, Joseph P. Y. Kao

**Affiliations:** 1 Center for Biomedical Engineering and Technology, University of Maryland School of Medicine, Baltimore, Maryland, United States of America; 2 Department of Physiology, University of Maryland School of Medicine, Baltimore, Maryland, United States of America; 3 Program in Neuroscience, University of Maryland, Baltimore, Baltimore, Maryland, United States of America; 4 Department of Neurology, University of Maryland School of Medicine, Baltimore, Maryland, United States of America; 5 Department of Biology, University of Maryland, College Park, Maryland, United States of America; University of California Berkeley, UNITED STATES

## Abstract

Ncm, 6-nitrocoumarin-7-ylmethyl, is a photolabile protective group useful for making “caged” molecules. Ncm marries the reliable photochemistry of 2-nitrobenzyl systems with the excellent stability and spectroscopic properties of the coumarin chromophore. From simple, commercially available starting materials, preparation of Ncm and its caged derivatives is both quick and easy. Photorelease of Ncm-caged molecules occurs on the microsecond time scale, with quantum efficiencies of 0.05–0.08. We report the synthesis and physical properties of Ncm and its caged derivatives. The utility of Ncm-caged glutamate for neuronal photostimulation is demonstrated in cultured hippocampal neurons and in brain slice preparations.

## Introduction

Caged molecules are biologically inert, but photolabile, molecules that are rapidly transformed by photolysis into bioactive molecules. Photolytic generation of bioactive molecules is often referred to as “photorelease” or “uncaging.” Since the introduction of caged ATP by Kaplan and colleagues in 1978 [1], a substantial number of reports of the synthesis of caged biomolecules have appeared, and the technique of effecting concentration jumps of bioactive molecules by flash photolysis has seen increasingly wide application in biology. Progress in the field has received expert comment and review at regular intervals [2–10]. The 2-nitrobenzyl (Nb) photolabile protective group (or “cage”) has been the archetype for making caged compounds, but it is not ideal for several reasons. The absorbance of Nb peaks at ~260 nm, but UV light at wavelengths below 300 nm is damaging to cells, whose principal constituents, proteins and nucleic acids, absorb at ~280 nm and ~260 nm, respectively. In the mid-300-nm spectral region, where cells are largely transparent, and where common light sources such as the mercury arc lamp and the argon ion laser emit, the molar absorptivity of Nb is low (200–350 M^-1^cm^-1^). Moreover, the side product of Nb photolysis, 2-nitrosobenzaldehyde [11, 12], is a reactive entity that can be injurious to cells.

One way to improve the photochemical characteristics of Nb is to enhance the ability of the chromophore to absorb light by introducing auxochromic substituents onto the aromatic ring. Methoxy is the most common auxochromic substituent for Nb. We and others have investigated the use of methoxy-substituted Nb to make caged derivatives of diverse biomolecules [13–25]. In the present study, we explored a different strategy, where the nitrobenzyl ring is fused to α-pyrone to generate a nitrocoumarin chromophore. The utility of the resulting photolabile group, 6-nitrocoumarin-7-ylmethyl (Ncm), in the preparation of caged molecules was assessed through the preparation and characterization of a series of derivatives of three amino acids that function as neurotransmitters—glycine, γ-aminobutyric acid, and L-glutamic acid. In these derivatives, the Ncm cage is covalently linked either to the α-amino group or to a carboxyl group. Derivatives that exhibited good physicochemical properties were characterized further in electrophysiological experiments on cultured neurons and on brain slices. The use of Ncm to cage a D-amino acid, namely D-aspartate, to produce a caged molecule that does not activate fast glutamate receptors, but that is a substrate for glutamate transporters, has been reported. [26]

## Materials and Methods

### Chemical Synthesis

Reagents and solvents, of ACS or higher grade, were purchased from commercial suppliers and used without further purification. NMR spectra were recorded on a 300 MHz instrument (QE-300, General Electric, Fairfield, CT) or on a 500 MHz instruments (INOVA, Varian Instruments, Palo Alto, CA). NMR spectra recorded in CDCl_3_ and DMSO-*d*_6_ were referenced to Me_4_Si; those recorded in D_2_O were referenced to the residual solvent peak (δ_H_ 4.80). Coupling constants are given in hertz (Hz). Following abbreviations were used to explain the multiplicity of NMR signals: s = singlet; d = doublet; dd = doublet of doublets; t = triplet; m = multiplet. Column chromatography was on silica gel (200–240 mesh, EM Science, Gibbstown, NJ). Analytical and preparative separations were performed on an HPLC system (Model 600, Waters Corp., Milford, MA) fitted with a photodiode array detector and a semi-preparative reverse phase column (Intersil 5 μm, MetaChem Technologies, Inc., Torrance, CA). A binary solvent system was used for HPLC: Solvent A, 0.1% (v/v) trifluroacetic acid in water; Solvent B, acetonitrile. Melting points were determined on a model Mel-Temp II apparatus (Laboratory Devices, Holliston, MA) and are uncorrected. Absorption spectra were acquired on a UV-visible spectrophotometer (Cary 300, Varian Instruments). HRMS (EI and FAB) analyses were performed at the mass spectrometry facility in the Biochemistry Department at Michigan State University (East Lansing, MI). Steady-state photolysis of samples was achieved with the multi-line UV emission of an argon ion laser (BeamLok 2065-7S, Spectra Physics, Santa Clara, CA).

#### 7-Methyl-6-nitrocoumarin (1)

7-methylcoumarin (10.0 g, 62.4 mmol) was dissolved in 60 mL conc. H_2_SO_4_. To the stirred solution maintained in an ice/water bath, KNO_3_ (7.57 g, 74.9 mmol) was added in portions over 15 min. After being stirred for 1 h at ice/water temperature, the reaction mixture was poured into 2000 mL of ice/water mixture, and the solid precipitate was collected. The preparative procedure was repeated once and the combined crude product was washed repeatedly with water and dried in air. Recrystallization from 95% ethanol afforded **1** (15.9 g, 62%): mp 198–199°C; ^1^H NMR (CDCl_3_) δ = 8.24 (s, 1H), 7.73 (d, 1H, *J* = 9.5 Hz), 7.28 (s, 1H), 6.51 (d, 1H, J = 9.5 Hz), 2.72 (s, 3H); *m/z* (‒ESI) 204.0301 ((M‒H)^‐^, C_10_H_6_NO_4_ requires 204.0297).

#### 7-Bromomethyl-6-nitrocoumarin (2)

Compound **1** (3.00 g, 14.6 mmol), benzoyl peroxide (0.88 g, 3.6 mmol), and NBS (5.20 g, 29.2 mmol) were added to a round bottom flask. The flask was evacuated for 10 min and filled with dry argon; the purge procedure was repeated twice more. Dry CCl_4_ (200 mL) was added to the flask and the reaction mixture was refluxed for 24 h. Thereafter, more pre-dried NBS (5.20 g 29.2 mmol) and benzoyl peroxide (0.88 g, 3.6 mmol) were added and reflux was continued for 6 h. The reaction mixture was filtered, and solvent removal under vacuum yielded the crude material, which was purified by flash chromatography. Compounds **1** and **2** co-eluted under all solvent conditions investigated. With CH_2_Cl_2_ as eluant, **2** was obtained in 80% purity (1.8 g; 34% yield), the balance being **1**. Compounds **1** and **2** could be separated by preparative HPLC, with the following percentage profiles of solvents A and B: 50:50 for 20 min, 0:100 for 5 min, and 50:50 for 5 min. Under these conditions **1** and **2** had retention times of 11.6 and 14.4 min, respectively. Appropriate fractions were pooled and reduced to dryness under vacuum to yield pure **2**: mp 174–176°C; ^1^H NMR (CDCl_3_) 8.30 (s, 1H), 7.75 (d, 1H, *J* = 9.7 Hz), 7.51 (s, 1H), 6.58 (d, 1H, *J* = 9.7 Hz), 4.90 (s, 2H); *m/z* (EI) 282.8482 (M^+^, C_10_H_6_BrNO_4_ requires 282.9480).

Routinely, flash column chromatography of the crude product afforded **2** whose purity was in the range of 70 ‒ 95% (the balance being **1**). Since compound **1** in the mixture is unreactive in alkylation reactions, the mixture can be used directly in the preparation of compounds **4**–**12**. The relative proportion of **2** and **1** in the mixture can be determined by integration of peaks in the ^1^H NMR spectrum at δ = 4.90 and 2.72, respectively.

#### *N*-[(6-nitrocoumarin-7-yl)methyl]-L-glutamic acid di-*t*-butyl ester (3)

Compound **2** (1 g, 90% purity, 3.1 mmol), H-Glu(O*t*Bu)-O*t*Bu.HCl (1.04 g, 3.5 mmol) and Et_3_N (1.4 mL, 10 mmol) were dissolved in 6 mL of dry DMSO. The mixture was stirred at room temperature for 4.5 h. Et_3_N and DMSO were removed under vacuum and the resulting residue was purified by column chromatography (5–10% CH_3_CN/CH_2_Cl_2_) to yield *N*-[(6-nitrocoumarin-7-yl)methyl]-L-glutamic acid, di-t-butyl ester **3** as a viscous oil (0.83 g, 57%). ^1^H NMR (CDCl_3_) 8.22 (s, 1H), 7.77 (d, 1H, *J* = 9.5 Hz), 7.72 (s, 1H), 6.54 (d, 1H, *J* = 9.5 Hz), 4.14 (dd, AB type, 2H, *J*_AB_ = 16 Hz), 3.15 (m, 1H), 2.38 (m, 2H), 1.98–1.82 (m, 2H), 1.47 (s, 9H), 1.45 (s, 9H).

#### *N*-[(6-nitrocoumarin-7-yl)methyl]-L-glutamic acid (4, *N*-Ncm-Glu)

Compound **4** was prepared following our previously reported procedure. [27] A stirred solution of **3** (0.10 g, 0.19 mmol) in 1 mL glacial HOAc was maintained in an ice/water bath. Concentrated HBr (1 mL) was added and stirring was continued for 15 min. Removal of HOAc and HBr under vacuum yielded a residue, which was dissolved in 1.2 mL water and purified by HPLC (CH_3_CN-H_2_O containing 0.1% v/v TFA: 10% to 100% CH_3_CN over 16 min.). The desired product eluted with a retention time of 10–12 min. The eluted fractions were pooled and lyophilized to yield a white powder (52 mg, 68%). ^1^H DMSO-*d*_6_ 8.49 (s, 1H), 8.15 (d, 1H, *J* = 9.8 Hz), 7.71 (s, 1H), 6.63 (d, 1H, *J* = 9.5Hz), 4.05 (dd, AB type, 2H, *J*_AB_ = 15.3 Hz), 3.17 (m, 1H), 2.32–2.29 (m, 1H), 1.86–1.80 (m, 1H), 1.76–1.66 (m, 1H); *m/z* (FAB) 351.0829 (MH^+^, C_15_H_15_N_2_O_8_ requires 351.0828).

#### *N*-[(6-nitrocoumarin-7-yl)methyl]glycine *t*-butyl ester (5)

To a solution of **2** (0.20 g, 92% purity, 0.64 mmol) and H-Gly-OBu-*t*.HCl (0.13 g, 0.77 mmol) in 3 mL DMSO, Et_3_N (0.39 mL, 2.8 mmol) was added. The reaction mixture was stirred at room temperature for 4 h. DMSO and excess Et_3_N were removed under vacuum. The resulting solid residue was purified by column chromatography (10–25% CH_3_CN in CH_2_Cl_2_) to yield *N*-[(6-nitrocoumarin-7-yl)methyl]glycine *t*-butyl ester **5** (0.11 g, 51%): ^1^H NMR (CDCl_3_) δ = 8.24 (s, 1H), 7.77–7.72 (m, 2H), 6.54 (d, 1H, *J* = 9.5 Hz), 4.22 (s, 2H), 3.35 (s, 2H), 1.46 (s, 9H).

#### *N*-[(6-nitrocoumarin-7-yl)methyl]glycine (6, *N*-Ncm-Gly)

TFA (3 mL) was added to a stirred solution of *t*-butyl ester **5** (0.11 g 0.33 mmol) in dry CH_2_Cl_2_ (3 mL). After 2.5 h at room temperature, the reaction mixture was reduced to dryness under vacuum. The residue was purified by column chromatography (10% HOAc, 10–20% MeOH in CH_2_Cl_2_) to give **6** as an acetate salt. (0.09 g, 80% yield). ^1^H NMR (DMSO-*d*_6_) δ = 8.47 (s, 1H), 8.12 (d, 1H, *J* = 9.5 Hz), 7.67 (s, 1H), 6.59 (d, 1H, *J* = 8.7 Hz), 4.01 (s, 2H), 2.92 (s, 2H), 1.81 (s, 3H). To obtain a crystalline product, the acetate salt of **6** was converted to the HCl salt by dissolving the acetate salt in 10 mL of 0.1% HCl and then dried by lyophilization (0.080 g, 77%); *m/z* (FAB) 279.0616 (MH^+^, C_12_H_11_N_2_O_6_ requires 279.0617).

#### *N*-[(6-nitrocoumarin-7-yl)methyl]-4-aminobutyric acid *t*-butyl ester (7)

To a solution of of **2** (0.25 g, 70% purity, 0.61 mmol) and H-Abu-OBu-*t*.HCl (0.17 g, 0.86 mmol) in 5 mL of dry CH_3_CN, Et_3_N (0.24 mL, 1.7 mmol) was added. The stirred mixture was kept in an ice/water for 45 min. Thereafter, the solvent was removed under vacuum and the resulting residue was purified by column chromatography (5% MeOH, 20% CH_3_CN, CH_2_Cl_2_) to give *N*-[(6-nitrocoumarin-7-yl)methyl]-4-aminobutyric acid *t*-butyl ester **7** as a thick, yellow oil (0.13 g, 59%). ^1^H NMR (CDCl_3_) 8.22 (s, 1H), 7.77–7.72 (m, 2H), 6.53 (d, 1H, *J* = 9.7 Hz), 4.17 (s, 2H), 2.67 (t, 2H, *J* = 6.6 Hz), 2.31 (t, 2H, *J* = 7.0 Hz), 1.81 (t, 2H, *J* = 7.0 Hz), 1.44 (s, 9H).

#### *N*-[(6-nitrocoumarin-7-yl)methyl]-4-aminobutyric acid (8, *N*-Ncm-GABA)

Trifluoroacetic acid (3 mL) was added to a solution of compound **7** (0.13 g) in 3 mL dry CH_2_Cl_2_; the resulting solution was stirred for 2.5 h at room temperature and then reduced to dryness under vacuum. Chromatography of the residue on silica gel (10% HOAc and 10% MeOH in CH_2_Cl_2_) yielded the acetate salt of **8**, which was dissolved in 0.1% HCl (10 mL) and lyophilized to yield the HCl salt (0.12 g, 97%). ^1^H NMR (D_2_O) 8.68 (s, 1H), 8.11 (d, 1H, *J* = 9.5 Hz), 7.71 (s, 1H), 6.71 (d, 1H, *J* = 9.7), 4.63 (s, 2H), 3.33 (t, 2H, *J* = 6.6 Hz), 2.55 (t, 2H, *J* = 7.0 Hz), 2.07 (t, 2H, *J* = 6.8 Hz); *m/z* (FAB) 307.0931 (MH^+^, C_14_H_15_N_2_O_6_ requires 307.0930).

#### *N*-(*t*-butoxycarbonyl)-L-glutamic acid 1-*t*-butyl-5-(6-nitrocoumarin-7-yl)methyl ester (9)

Et_3_N (0.34 mL, 2.4 mmol) was added to a solution of **2** (0.35 g, 70% purity, 0.86 mmol) and *N*-Boc-Glu-O*t*Bu (0.37 g, 1.2 mmol) in dry DMSO (4 mL). The mixture was stirred at room temperature for 5.5 h and then reduced to dryness under vacuum. Chromatography on silica gel (5–10% CH_3_CN in CH_2_Cl_2_) yielded *N*-(*t*-butoxycarbonyl)-L-glutamic acid 1-*t*-butyl-5-(6-nitrocoumarin-7-yl)methyl ester **9** (0.33 g, 76%). ^1^H NMR (CDCl_3_) 8.38 (s, 1H), 7.77 (d, 1H, *J* = 9.7 Hz), 7.56 (s, 1H), 6.57 (d, 1H, *J* = 9.7 Hz), 5.62 (m, 2H), 5.11 (m, 1H), 4.25 (m, 1H), 2.62–2.53 (m, 2H), 2.22 (m, 1H), 1.94 (m, 1H), 1.48 (s, 9H), 1.45 (s, 9H).

#### L-Glutamic acid 5-(6-nitrocoumarin-7-yl)methyl ester (10, γ-*O*-Ncm-Glu)

Trifluoroacetic acid (3 mL) was added to a solution of compound **9** (0.25 g, 0.49 mmol) in dry CHCl_3_ (3 mL). After stirring for 21 h at room temperature, the solution was reduced to dryness under vacuum. Chromatography of the residue (10–20% HOAc, 10–20% MeOH in CH_2_Cl_2_) yielded the acetate salt of **10** (0.18 g, 90%). NMR analysis of this compound was hampered by the partial conversion of **10** to compound **13** in DMSO-*d*_6_, presumably caused by the trace of H_2_O present in DMSO-*d*_6_. The NMR spectrum initially showed the presence of both **10** and **13** (as a minor component). After storage of the DMSO-*d*_6_ sample in the dark for 48 h, conversion of **10** to **13** was complete, as verified by NMR spectroscopy.

#### *N*-(*t*-butoxycarbonyl)glycine (6-nitrocoumarin-7-yl)methyl ester (11)

Compound **2** (0.3 g, 70% purity, 0.73 mmol), *N*-Boc-Gly (0.18 g, 1.02 mmol), and Et_3_N (0.59 mL, 4.2 mmol) were dissolved in dry DMSO (3 mL). The mixture was stirred at room temperature for 3 h and then reduced to dryness under vacuum. The residue was chromatographed (5–20% CH_3_CN in CH_2_Cl_2_) to give *N*-(*t*-butoxycarbonyl)glycine (6-nitrocoumarin-7-yl)methyl ester **11** (0.23 g, 83%). ^1^H NMR (CDCl_3_) 8.41 (s, 1H), 7.80 (d, 1H, *J* = 9.5 Hz), 7.58 (s, 1H), 6.58 (d, 1H, *J* = 9.5 Hz), 5.68 (s, 2H), 5.14 (broad s, 1H), 4.08 (d, 2H, *J* = 5.3), 1.47 (s, 9H).

#### Glycine (6-nitrocoumarin-7-yl)methyl ester (12, *O*-Ncm-Gly)

A solution of compound **11** (0.23 g, 0.60 mmol) in EtOAc (10 mL) was mixed with 10 mL of EtOAc saturated with HCl gas and stirred for 30 min at room temperature. Solvent removal under vacuum left a residue that was purified by column chromatography (10% HOAc, 10–20% MeOH in CH_2_Cl_2_) to give the acetate salt of compound **12** (0.1 g, 48%). ^1^H DMSO-*d*_6_ 8.69 (s, 1H), 8.20 (d, 1H, *J* = 9.2 Hz), 7.68 (s, 1H), 6.68 (d, 1H, *J* = 9.2 Hz), 5.54 (s, 2H), 3.60–3.43 (m, 2H), 1.84 (s, 3H).

#### 7-Hydroxymethyl-6-nitrocoumarin (13)

The acetate salt of compound **12** (25 mg, 0.07 mmol) was dissolved in 20 mL of 0.1 M phosphate buffer (pH 7.4) and maintained in the dark for 36 h at room temperature. Thereafter, the solution was extracted with CH_2_Cl_2_ and the extract was dried under vacuum to yield a yellow solid (10 mg, 64%). ^1^H DMSO-*d*_6_ 8.60 (s, 1H), 8.18 (d, 1H, *J* = 9 Hz), 7.68 (s, 1H), 6.63 (d, 1H, *J* = 9.5 Hz), 5.79 (s, 1H; exchangeable with D_2_O), 4.90 (s, 2H); *m/z* (FAB) 222.0402 (MH^+^, C_10_H_8_NO_5_ requires 222.0403).

#### 7-Formyl-6-nitrosocoumarin (14)

7-Bromomethyl-6-nitrocoumarin (Compound **2**) (0.68 g, 2.4 mmol) was dissolved in 70% aqueous acetone (130 mL) and added dropwise over 75 min to a vigorously-stirred solution of AgNO_3_ (1.65 g, 9.7 mmol) in 50% aqueous acetone (100mL) maintained at 50°C. After addition was complete, stirring was continued for 1 h. Thereafter, the reaction mixture was filtered, and the solids were rinsed with minimal 50% aqueous acetone. Acetone was removed from the filtrate by rotary evaporation, and the remaining aqueous phase was extracted with ether (6 × 100 mL). The combined ether extract was washed with water (125 mL), dried over anhydrous Na_2_SO_4_, filtered, and reduced to dryness under vacuum. The residue was purified on silica gel (0.3:3.7 EtOAc:CH_2_Cl_2_) to yield compound **13** (51 mg, 0.23 mmol). Compound **13** (51 mg) was placed in a watch glass (9 cm diameter) and dissolved in ~7 mL acetonitrile to form a thin circular pool of solution ~5 cm in diameter; the solution was gently stirred magnetically. A UV lamp (UVGL-58, UVP, Inc., Upland, CA), delivering ~0.8 mW.cm^-2^ of 366-nm light, was positioned over the solution for 90 min. To maintain constant solution volume, acetonitrile was added as needed. Photolysis progress was monitored by TLC. To minimize oxidative decomposition of the nitroso product, the entire apparatus was immersed in a chamber purged with argon. Upon completion of photolysis, acetonitrile was evaporated under a gentle stream of N_2_, and the residue was purified on silica gel (1:3 EtOAc:hexanes; silica gel and solvents pre-equilibrated with N_2_) to yield nitroso aldehyde **14** as a pale yellow solid (20 mg, 98 μmol; 43%). ^1^H NMR DMSO-_*6*_ 11.58 (s, 1H), 8.23 (d, 1H, *J* = 10.2 Hz), 7.90 (s, 1H), 7.23 (s, 1H), 6.78 (d, 1H, *J* = 9.4 Hz); *m/z* (ESI) 204.0290 (MH^+^ C_10_H_6_NO_4_ requires 204.0297).

### Quantum Yield of Photolysis

The quantum yields for the photolysis of compounds **4**, **6** and **8** were determined by an HPLC-based method [28]. The frequency-tripled (355 nm) output of a pulsed Nd:YAG laser (QuantaRay GCR-18S, Spectra Physics Lasers, Santa Clara, CA) was isolated from the fundamental and second harmonic emissions by a pair of harmonic separators (BSR-35, CVI Laser, LLC, Albuquerque, NM) and used for photolysis; the energy at the sample was ~100 mJ per pulse. The light intensity was determined by potassium ferrioxalate actinometry [29], and showed the laser power to be highly stable and the integrated energy output to be a linear function of time. The photorelease reactions were characterized by simple 1:1 stoichiometry between the caged starting material and the released product. To quantitate disappearance of starting caged compound, equal amounts of 4-nitrophenol were added as an internal standard to irradiated samples and to unirradiated controls. In the HPLC traces of these samples, the ratio of the areas of the peaks corresponding to the starting material and the internal standard were determined. From this, the loss of the starting material on photolysis was calculated. In all cases, 0.1 M sodium phosphate buffer (pH 7.41) was used as solvent, and the extent of photolytic conversion was kept at ~10%. Each photolysis experiment was performed in triplicate, and each of the triplicate samples was analyzed two or three times by HPLC.

### Reaction of Nitroso Aldehyde with Glutathione

The absorbance of a solution of 25 μM nitroso aldehyde **14** in 10 mM sodium phosphate buffer (pH 7.0, 24°C) was recorded. Thereafter, glutathione (50 μM disodium salt) was added. After rapid mixing, the absorbance of the solution at 328 nm was sampled and recorded at 20 Hz. Data acquisition was terminated when the absorbance had attained a constant value. The absorbance at time *t*, *A*(*t*), was subtracted from the mean baseline absorbance, *A*_0_, to yield *ΔA*(*t*). The *ΔA*(*t*) data were analyzed by nonlinear least-squares curve fitting with the following function:

$$y=y_{0}+S\left[1+\frac{1}{e^{-k_{\text{app}}t}-2}\right]$$

where *y*_0_, *S*, and *k*_app_ are fitting parameters. Specifically, *k*_app_ = 0.5[GSH]_0_*k*, where *k* is the bimolecular rate constant at pH 7.0 and 24°C, and [GSH]_0_ is the GSH concentration at *t* = 0; *y*_0_ and *S* are baseline and scaling parameters, respectively. Details of the derivation are in S1 Text.

### Kinetics of Flash Photolysis

Flash photolysis kinetics were investigated as previously described [30]. Briefly: A solution of caged compound in a fused silica cuvette was positioned in a flash photolysis kinetic spectrophotometer (LP920, Edinburgh Instruments, Livingston, UK) and was magnetically stirred at room temperature. Samples were equilibrated with air. Photolysis of the sample was effected by an 8.6-ns (FWHM), 100-mJ pulse of 355-nm (third harmonic) light from a Nd-YAG laser (Quanta-Ray GCR-18S, Spectra Physics). A pair of harmonic separators (BSR-35, CVI Laser, LLC) was used to remove residual fundamental (1064 nm) and second harmonic (532 nm) laser emissions from the photolysis beam. A probe beam from a stabilized xenon arc lamp was focused so that the focal point coincided with the region in the sample where maximal photolysis occurred. The spectrum of the probe beam was obtained through a monochromator. Modulation of the probe beam intensity by a transient photochemical intermediate species was monitored at a chosen wavelength by a fast photomultiplier tube. The intensity-vs-time data were converted into absorbance-vs-time traces. Exponential decay times were extracted from the data through nonlinear least-squares analysis. Data in the initial 50-ns window contain artefactual contributions from scattering of the laser pulse and instabilities in the high-sensitivity detection circuitry, and are not included in data analysis.

### HPLC Analysis of Free Amino Acids

Analysis of free amino acids by HPLC was performed according to published procedures [31]. Calibration data for determining the limit of detection are provided as S1 Fig.

### Responses Evoked in Cultured Hippocampal Neurons by Photolysis of Caged Glutamate

Recording of ionic currents evoked in cultured hippocampal neurons by photolysis of caged glutamates was performed essentially as described previously [32]. All procedures were approved by the Institutional Animal Care and Use Committee at the University of Maryland School of Medicine. Briefly: Neurons were dissociated from the hippocampi of 20-day-old rat embryos, plated onto acid-washed, collagen-coated glass coverslips (25 mm diameter, thickness No. 1), and maintained in culture for 2–3 weeks[33]. Electrophysiological measurements were performed with a patch-clamp amplifier (model 3900, Dagan Corp. Minneapolis, MN) and borosilicate microelectrodes (3–5 MΩ resistance) in the whole-cell configuration, with membrane potential voltage-clamped at -80 mV. Microelectrode internal solution contained (in mM) 140 CsCl, 5 EGTA, 2 CaCl_2_, 1 MgCl_2_, and 10 HEPES (adjusted to pH 7.3 with CsOH); extracellular solution contained (in mM) 150 NaCl, 3 KCl, 2 CaCl_2_, 1 MgCl_2_, and 10 HEPES (adjusted to pH 7.3 with NaOH). Caged glutamate (1 mM *N*-Ncm-Glu or *N*-Nmoc-Glu [32]) was delivered through the extracellular solution. For photolysis of caged glutamate, the UV output (351–364 nm) of an argon ion laser (Stabilite 2017, Spectra Physics, Mountain View, CA) was launched into a 25-μm-diameter quartz optical fiber; the power at the exit end of the fiber was ~5 mW. A relay lens system and a dichroic mirror were used to project the image of the exit face of the optical fiber onto an optical plane that is conjugate to the field diaphragm, along the epifluorescence path of the inverted microscope (Diaphot, Fluor ×40 objective, N.A. 1.3, Nikon). This produced a photolysis spot ~9 μm in diameter, which was positioned over the cell body; power at the sample was estimated to be 1–1.5 mW, or an irradiance of 16–24 μW/μm^2^). Duration of photolytic light pulses was controlled by a laser shutter (NM Laser Products, Sunnyvale, CA). Shutter gating and electrophysiological data acquisition were controlled through pClamp software (Axon Instruments, Sunnyvale, CA).

### Responses Evoked by Glutamate Photorelease in the Mouse Auditory Cortex

All procedures followed the University of Maryland College Park animal care and use regulations. In acute mouse brain slices, responses of auditory cortex (ACX) neurons to photorelease of *N*-Ncm-Glu were recorded as intracellular Ca^2+^ signals reported by Oregon Green 488 BAPTA-1, a fluorescent Ca^2+^ indicator. The acetoxymethyl (AM) ester form of the indicator (Oregon Green 488 BAPTA-1 AM, Invitrogen; 50 μg) was first dissolved in 4 μl of 20% (w/v) Pluronic F-127 in DMSO and then diluted with 16–28 μl artificial cerebrospinal fluid (ACSF) containing (in mM): 130 NaCl, 3 KCl, 1.25 KH_2_PO_4_, 20 NaHCO_3_, 10 glucose, 1.3 MgSO_4_, 2.5 CaCl_2_ (pH 7.35–7.4 in equilibration with 95% O_2_−5% CO_2_). The ACSF contained, in addition, either 100 μM AlexaFluor 594 (for visualization) or SR-101 (for visualization and astrocyte identification). Pipettes (2–4 μm tip diameter) were pulled (Sutter P2000), filled with the above dye solution and introduced into the cortex. The pipette tip was visualized under 2-photon scanning and gradually advanced into the cortex. At a depth of 350–500 μm the dye solution was gradually ejected with 5–20 psi (PV830 Pneumatic PicoPump, WPI) pressure pulses (pulse duration 0.2–1 s, 50–130 pulses total over 15 min). After 2 h animals were deeply anesthetized with isoflurane. A block of brain containing ACX was removed and slices (350–400 μm thick) were cut under ice-cold ACSF on a vibrating microtome (Leica). Thereafter, the slices were incubated in ACSF for 1 h at 30C and then at room temperature. For recording, a slice was held in a chamber under the microscope and superfused (2–4 mL.min^-1^) with ACSF at room temperature. *N*-Ncm-Glu (0.5–1 mM) was introduced through the ACSF superfusate. The 355-nm emission from a quasi-continuous laser (model 3505, 100 kHz pulse rate, DPSS Lasers, Inc., Santa Clara, CA, 2–50 ms pulses) was launched into an optical fiber (25 μm core diameter, Oz Optics, Ontario, Canada); at the distal end, power was ≤ 50 mW. Light exposure (≤ 50 ms) was controlled by gating the laser output with a shutter (NM Laser Products). The distal end of the fiber was held within a bent glass pipette to reduce beam elongation and to deliver light to a small area. In preliminary experiments we verified that the power used provided reliable direct activation of neurons recorded in whole-cell patch configuration only in the presence of caged glutamate. For imaging, the brain slice was illuminated at 488 nm (Lamda LS, Sutter Instruments) and fluorescence images were acquired with a cooled CCD camera (CoolSnap HQ2, Roper Scientific, Tucson, AZ).

### Data Analysis and Presentation

Numerical data analysis was performed using OriginPro (OriginLab, Northampton, MA) or MATLAB 7.10 (MathWorks, Natick, MA); MATLAB was used for image analysis.

## Results and Discussion

### Chemical Synthesis

**Fig 1:**
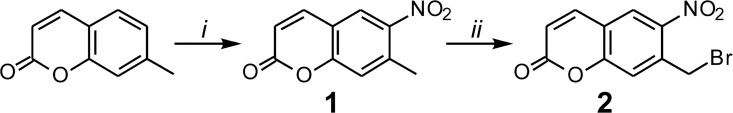
Synthesis of the caging reagent: Ncm bromide. Reagents and conditions: *i*) KNO_3_, concentrated H_2_SO_4_, 0°C, 1 h; *ii*) NBS, benzoyl peroxide, CCl_4_, reflux, 30 h.

**Fig 2:**
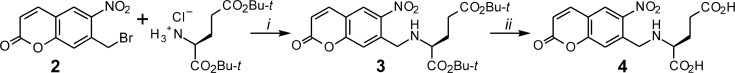
Synthesis of *N*-Ncm-Glu (glutamate caged on the α-amino group). Reagents and conditions: *i*) Et_3_N, DMSO, rt, 4.5 h; *ii*) HBr/HOAC, 0°C, 15 min.

**Fig 3:**
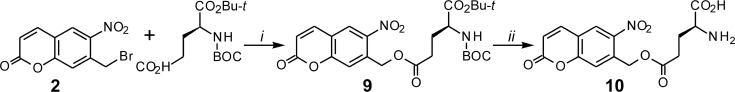
Synthesis of γ-*O*-Ncm-Glu (glutamate caged on the γ-carboxyl group). Reagents and conditions: *i*) Et_3_N, DMSO, rt, 5.5 h; *ii*) TFA, CHCl_3_, rt, 21 h.

**Fig 4:**
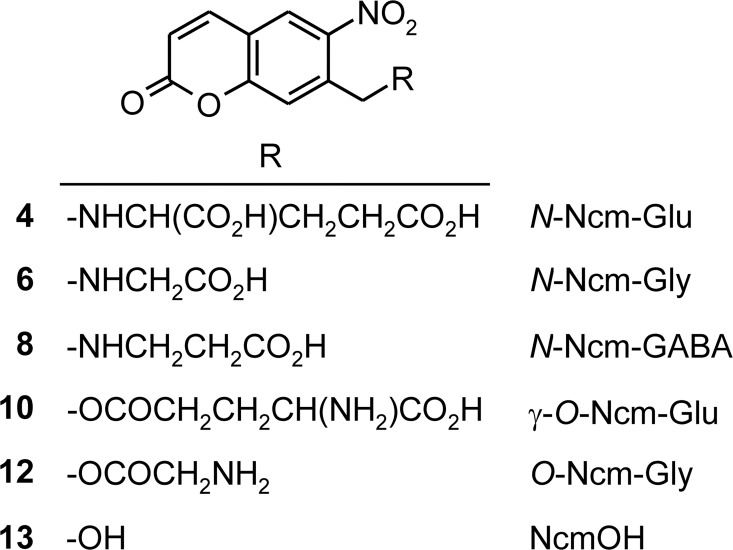
Summary of Ncm derivatives.

As shown in Fig 1, the activated photolabile protective group, 7-bromomethyl-6-nitrocoumarin (**2**) is readily made by regioselective nitration of 7-methylcoumarin with KNO_3_ in H_2_SO_4_ [34], followed by benzylic bromination with NBS. Fig 2 shows the preparation of a caged L-glutamic acid (**4**), where the Ncm moiety is linked to the α-amino group. L-glutamic acid di-*t*-butyl ester was *N*-alkylated with reagent **2**, followed by deprotection of the *t*-butyl esters with HBr in acetic acid. *N*-Ncm glycine (**6**) and *N*-Ncm-γ-aminobutyric acid (**8**) were prepared analogously. Fig 3 shows the preparation of a caged L-glutamic acid (**10**) wherein the photolabile Ncm group is linked to the γ-carboxyl group. *N*-Boc-L-glutamic acid 1-*t*-butyl ester was *O*-alkylated with reagent **2**, followed by removal of Boc and *t*-butyl protective groups with TFA in chloroform. *O*-Ncm glycine (**12**) was prepared analogously. A summary of all the Ncm-protected amino acid derivatives is shown in Fig 4.

### Stability of Ncm-caged Amino Acids at Physiological pH in the Absence of Light

In order for a caged amino acid to be useful in biological experiments, it must be stable in aqueous solution at physiological pH and not spontaneously cleave to yield the free amino acid. To assess chemical stability, solutions of compounds **4**, **6**, **8**, **10** and **12** in 0.1 M sodium phosphate buffer (pH 7.4) were maintained in the dark at room temperature (22 ± 1°C). Samples were drawn at various times for HPLC analysis. As expected, caged amino acids **4**, **6** and **8** (*N*-Ncm-Glu, -Gly, and–GABA, respectively), whose amino groups were protected with the Ncm group, did not spontaneously liberate free amino acids at pH 7.4 at room temperature, as verified by HPLC amino acid analysis. For example, in a 0.31 mM solution of *N*-Ncm-Glu in 0.1 M phosphate buffer (pH 7.4) maintained at 22°C for 25 hr, the upper limit of the amount of free glutamate present was determined by HPLC to be < 0.06 mole–%. The *N*-caged amino acids are thus very stable.

Whereas the *N*-caged amino acids showed high chemical stability, the *O*-caged compounds *O*-Ncm-Gly (**12**) and γ-*O*-Ncm-Glu (**10**)‒glycine and glutamate whose α and γ carboxyls, respectively, were esterified with Ncm‒were susceptible to spontaneous hydrolysis. The appearance of the hydrolysis product NcmOH (compound **13**) followed single-exponential kinetics and showed that *O*-Ncm-Gly hydrolyzed with a half-life of *t*_1/2_ = 6.54 ± 0.95 h, while γ-*O*-Ncm-Glu exhibited *t*_1/2_ = 54.1 ± 3.6 h (details in S2 Fig and S3 Fig). These results imply that at pH 7.4 at room temperature, samples of *O*-Ncm-Gly and γ-*O*-Ncm-Glu would liberate 1% free amino acid in 5.7 min and 47 min, respectively. This level of hydrolysis is likely to be unacceptable for most biological experiments. The *O*-caged amino acids are thus not expected to be useful for biological experiments.

Rapid spontaneous hydrolysis of an α-amino ester, as observed for *O*-Ncm-Gly, is not unusual, and has been observed in many systems [35–42]. A plausible explanation is neighboring group participation: The α-amino/ammonio group can perform intramolecular general acid-base catalysis and can stabilize the tetrahedral intermediate resulting from nucleophilic attack on the carbonyl [36, 43]. This view rationalizes the observation that β-amino esters hydrolyze more slowly than their α counterparts [44], and that *O*-Ncm-Gly hydrolyzes more quickly than γ-*O*-Ncm-Glu.

**Fig 5:**
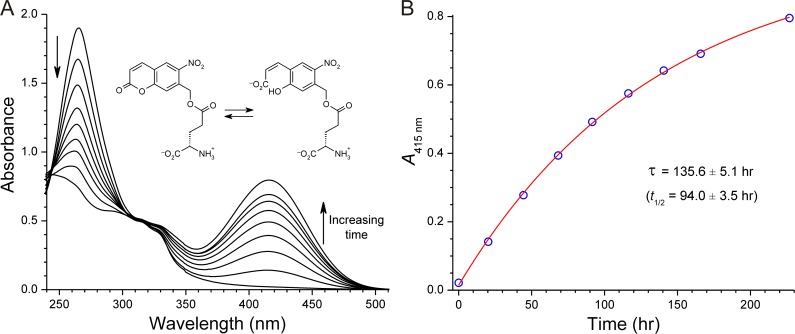
Ring-opening of the Ncm chromophore in *N*-Ncm-Glu. (A) UV-visible absorption spectra of a 83 μM solution of *N*-Ncm-Glu in 0.1 M sodium phosphate (pH 7.4) at 22°C. Spectra were recorded at the following times: 0, 20.3, 44.4, 68.2, 91.6, 116.3, 140.6, 165.9, 226.8 hours. Inset shows interconversion of the open and closed forms (H_2_O and H^+^ required for balance are omitted for visual clarity). (B) The increase of absorbance at 415 nm is well fit by a single exponential function.

The coumarin moiety of the Ncm chromophore could exist in the ring-opened form in aqueous solution. Indeed, on standing at 22°C, initially colorless solutions of the *N*-protected compounds **4**, **6** and **8** in 0.1 M phosphate buffer (pH 7.4) very slowly became tinged with yellow—suggestive of the presence of the ring-opened nitrophenolate form. The appearance of the long-wavelength absorption can be monitored spectroscopically. Fig 5A shows a series of UV-visible absorption spectra recorded over several days from a solution of *N*-Ncm-Glu (**4**) in 0.1 M phosphate buffer (pH 7.4) at 22°C. These spectra show a gradual diminution of the peak at 265 nm with concomitant growth of a new peak at 415 nm. Nonlinear least-squares analysis of these data show that, at pH 7.4 at room temperature (22°C), *N*-Ncm-Glu slowly ring-opens with a half-life of ~4 days (*t*_1/2_ = 94.0 ± 3.5 h; Fig 5B). As expected, ring opening is strongly temperature-dependent: at 4°C, the half-life lengthens markedly to 22.1 ± 4.1 days—more than sufficient for all biological experiments. Frozen aqueous solutions of *N*-Ncm-Glu stored at ‒20°C are stable for at least 17 months. The ring-opened form regenerates the intact Ncm chromophore under acidic conditions (S4 Fig). Importantly, the ring-opened form is not photosensitive and does not liberate free amino acid upon UV irradiation (see below).

### Physical Chemical Characterization of *N*-Ncm Amino Acids

**Fig 6:**
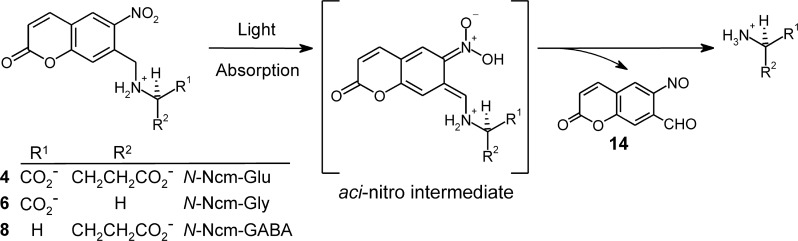
Photolysis of Ncm-caged amino acids. Light absorption leads to the transient *aci*-nitro intermediate, which decomposes into the free amino acid and the spent cage (7-formyl-6-nitrosocoumarin). The caged molecules are depicted as *N*-protonated species because the *pK*_a_s for the α-amino groups are all estimated to be higher than physiologic pH (details in S3 Text).

**Fig 7:**
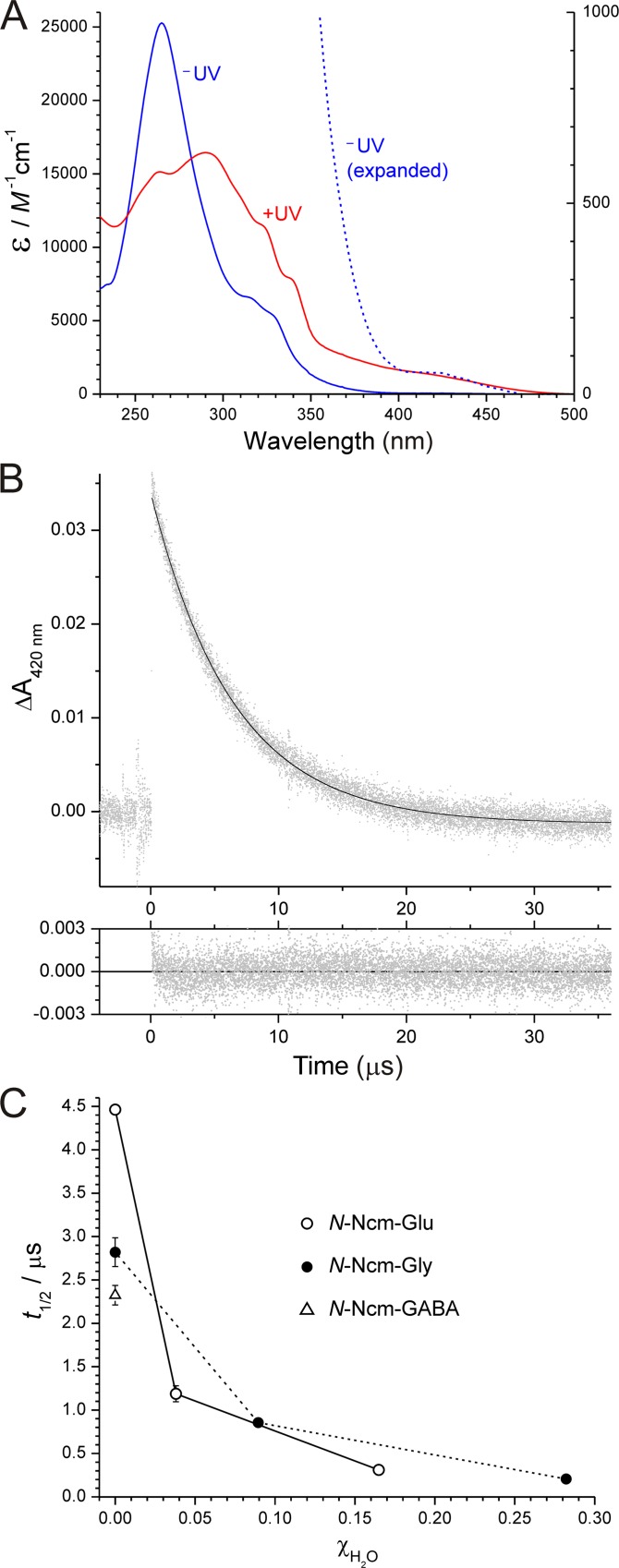
Physical chemical characterization of *N*-Ncm amino acids. (A) UV-visible absorption spectra of a solution of *N*-Ncm-Glu in 0.1 M phosphate buffer (pH 7.4) recorded before (blue solid line, labeled “‒UV”) and after (red solid line, labeled “+UV”) photolysis with 166 mW of UV light from an argon ion laser for 2 min. The blue dashed curve shows the long-wavelength (360–500 nm) region of the ‒UV curve on an expanded scale (refer to the *y*-axis scale on the right). (B) Transient absorbance change of a solution of *N*-Ncm-Glu in DMSO following photolysis with an 8.6-nsec pulse of 355-nm light delivered at time zero. In the upper panel, gray points are data and solid curve is a least-squares single-exponential fit to the data. The residuals of the fit are shown in the lower panel. (C) Dependence of photolysis kinetics on mole-fraction of water. The *t*_1/2_ values for transient spectral decay are plotted against mole fraction of water in DMSO for *N*-Ncm-Glu and *N*-Ncm-Gly. The *t*_1/2_ for *N*-Ncm-GABA in DMSO is also shown. Each point is the average of 3 replicate measurements; error bars represent standard deviations (where not seen, error bar is smaller than the symbol).

Physical chemical characterization was performed on the three *N*-Ncm-caged amino acids (**4**, **6**, and **8**). Photolysis of an *N*-Ncm-caged amino acid is represented in Fig 6. UV-visible absorption spectra of *N*-Ncm-Glu recorded before and after photolysis are shown in Fig 7; essentially identical spectra were recorded for *N*-Ncm-Gly and *N*-Ncm-GABA (not shown). The photolysis quantum yields were determined to be Φ = 0.054 ± 0.001 for *N*-Ncm-Glu (*n* = 3), Φ = 0.062 ± 0.007 for *N*-Ncm-Gly (*n* = 3), and Φ = 0.077 ± 0.001 for *N*-Ncm-GABA (*n* = 2), and have been corrected for the inner-filter effect of the photoproduct (see S2 Text for details). These quantum yields are very similar to those reported for other caged amino acids, which span the approximate range from 0.01 to 0.1 [8, 45–47].

To investigate the kinetics of photolysis, we used transient absorption spectroscopy to monitor the photolytically-generated short-lived *aci*-nitro intermediate [48–51] (Fig 6), the decay of which is commonly taken to be concomitant with cleavage of the protective group and release of product [52, 53]. In pure aqueous solvent, the photolysis kinetics of all three *N*-caged amino acids were too fast to be resolved in our flash photolysis apparatus. Therefore, we performed flash photolysis in DMSO with different mole fractions of water. The transient absorption trace recorded on photolysis of *N*-Ncm-Glu in DMSO by an 8.6-nsec pulse of 355-nm light is shown in Fig 7B. The decay followed a single-exponential time course with a time constant of τ = 6.43 ± 0.02 μsec, or equivalently, a half-life of *t*_1/2_ = 4.46 ± 0.01 μsec (n = 25 kinetic traces). The photolysis kinetics of *N*-Ncm-Gly and *N*-Ncm-GABA in DMSO were similar, being characterized by time constants of τ = 4.07 ± 0.14 μsec (*t*_1/2_ = 2.82 ± 0.10 μsec) and τ = 3.35 ± 0.08 μsec (*t*_1/2_ = 2.32 ± 0.05 μsec), respectively (n = 25 kinetic traces in each case). Photolysis kinetics accelerated with increasing mole fraction of water in the DMSO solution: for *N*-Ncm-Glu and *N*-Ncm-Gly, increasing the mole fraction of water from 0 to 0.16 and 0.28, respectively, accelerated the kinetics by more than 10-fold (Fig 7C). These results imply that in pure aqueous solution under flash photolysis conditions, release of free amino acids from the *N*-Ncm-caged amino acids should be complete within just a few μsec—much faster than is required in physiological experiments.

**Fig 8:**
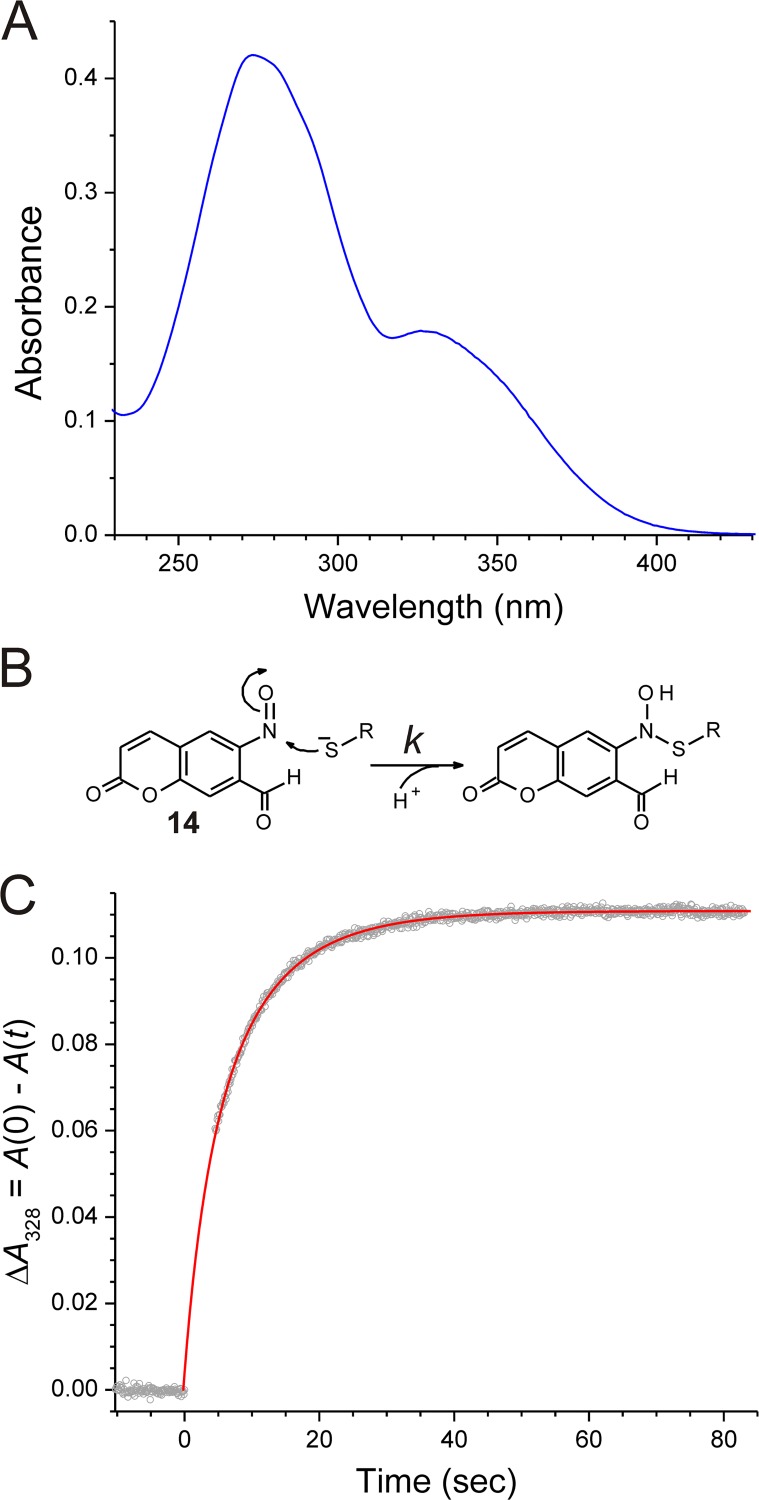
Nitroso photoproduct of the Ncm cage and its reaction with glutathione. (A) UV-visible spectrum of a 30 μM solution of 7-formyl-6-nitrosocoumarin (**14**) in 10 mM sodium phosphate buffer (pH 7). (B) Schematic representation of the reaction of **14** with a thiolate (RS^‒^), which adds nucleophilically to the nitroso group, and concomitantly disrupts the chromophore. The bimolecular rate constant is *k*. (C) Spectrophotometrically monitored time course of the reaction of **14** (25 μM) with glutathione (50 μM) in 10 mM sodium phosphate buffer (pH 7.0, 24°C). Grey circles are experimental data acquired at 20 Hz; red curve is the nonlinear least-squares fit to the function, $y=y_{0}+S[1+{\left(e^{-k_{\text{app}}t}-2\right)}^{-1}]$, where *k*_app_ = 0.5[GSH]_0_*k*, (*k* is the bimolecular rate constant indicated in panel B, and the initial glutathione concentration is [GSH]_0_ = 50 μM), and *y*_0_ and *S* are adjustable parameters (see Materials and Methods and S1 Text for details). For the data set shown, *k* = 3.74 × 10^3^ M^-1^s^-1^.

Photolysis of the Ncm cage is expected to generate an *o*-nitrosoaldehyde, namely 7-formyl-6-nitrosocoumarin, **14** (Fig 6). Compound **14** was purified from an exhaustively photolyzed solution of compound **13**. As expected from its more extended conjugation, compound **14** absorbs more strongly at longer wavelengths (330–410 nm) than the unphotolyzed Ncm cage (Fig 8A). Reaction of aryl nitroso compounds with thiols has been studied extensively [54–63], and is a likely cause of cytotoxicity. Reaction proceeds by nucleophilic addition of the thiolate to the nitroso to generate a common intermediate [64, 65], from which several pathways can lead to different final products [66]. Thiolate addition is accompanied by concomitant destruction of the nitroso chromophore (Fig 8B); the resulting absorbance change can be used to monitor reaction progress. A time course of the reaction of compound **14** with glutathione is shown in Fig 8C. Nonlinear least-squares analysis of the kinetic data yielded a value for the bimolecular rate constant: *k* = 3.86 (± 0.10) × 10^3^ M^-1^s^-1^ (*n* = 3) at pH 7.0 and 24°C. Comparison with published studies performed at pH 7.5 (after adjusting for the pH difference) indicates that the Ncm photoproduct is less reactive than 2-nitrosobenzaldehyde by more than 10-fold (see S4 Text for details of the quantitative comparison).

### Test of Ncm-caged Glutamate on Cultured Hippocampal Neurons

**Fig 9:**
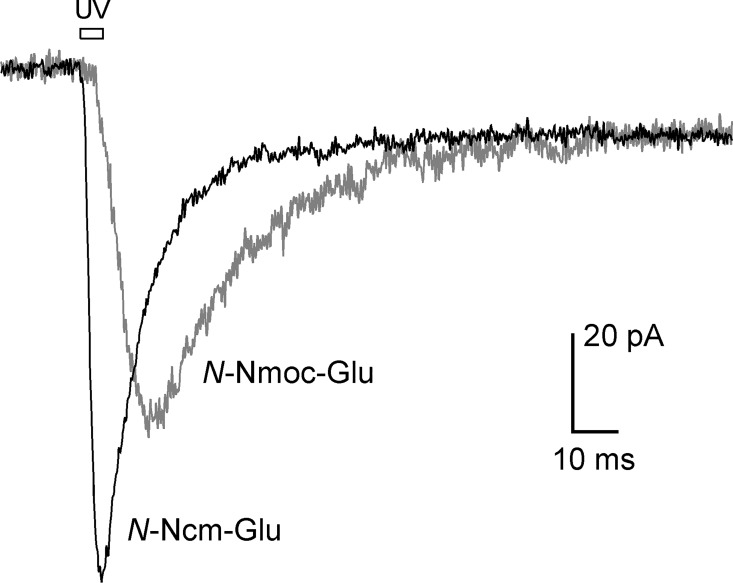
Comparison of ionic currents evoked by photoreleasing *N*-Ncm-Glu and *N*-Nmoc-Glu onto cultured hippocampal neurons. Bar above the trace marks the 5-ms duration of the UV laser pulse. Caged glutamate concentration was 1 mM. Currents shown are the average from 12 cells for *N*-Ncm-Glu and 7 cells for *N*-Nmoc-Glu.

We first tested the biological efficacy of *N*-Ncm-Glu on cultured rat hippocampal neurons, and compared its performance to a caged glutamate we had reported previously—*N*-(2-nitromandelyl)oxycarbonyl-L-glutamic acid (*N*-Nmoc-Glu) [32]. Photorelease of glutamate activates an inward ionic current in hippocampal neurons; this current can be readily monitored electrophysiologically under voltage-clamp conditions. Fig 9 shows inward currents in hippocampal neurons evoked by photorelease of *N*-Ncm-Glu and *N*-Nmoc-Glu. The inward current activated by *N*-Ncm-Glu photolysis developed more rapidly and had larger amplitude than that activated by *N*-Nmoc-Glu photolysis. Moreover, for *N*-Ncm-Glu, activation of inward current was immediate upon initiation of the UV light pulse, whereas for *N*-Nmoc-Glu, the current activated only after a latency of ~3 ms. The observed kinetic differences stem from the structural differences between the two caged glutamates. In *N*-Ncm-Glu, the photolabile group is directly attached to the α-amino group of glutamate; therefore photocleavage yields free glutamate directly. In contrast, in *N*-Nmoc-Glu, the photolabile 2-nitromandelyl moiety is attached to the α-amino group through a carbamate linkage; photocleavage leads to *N*-carboxyglutamate, which then decarboxylates to yield free glutamate [32].

### Test of Ncm-caged Glutamate on Acutely Prepared Brain Slices

**Fig 10:**
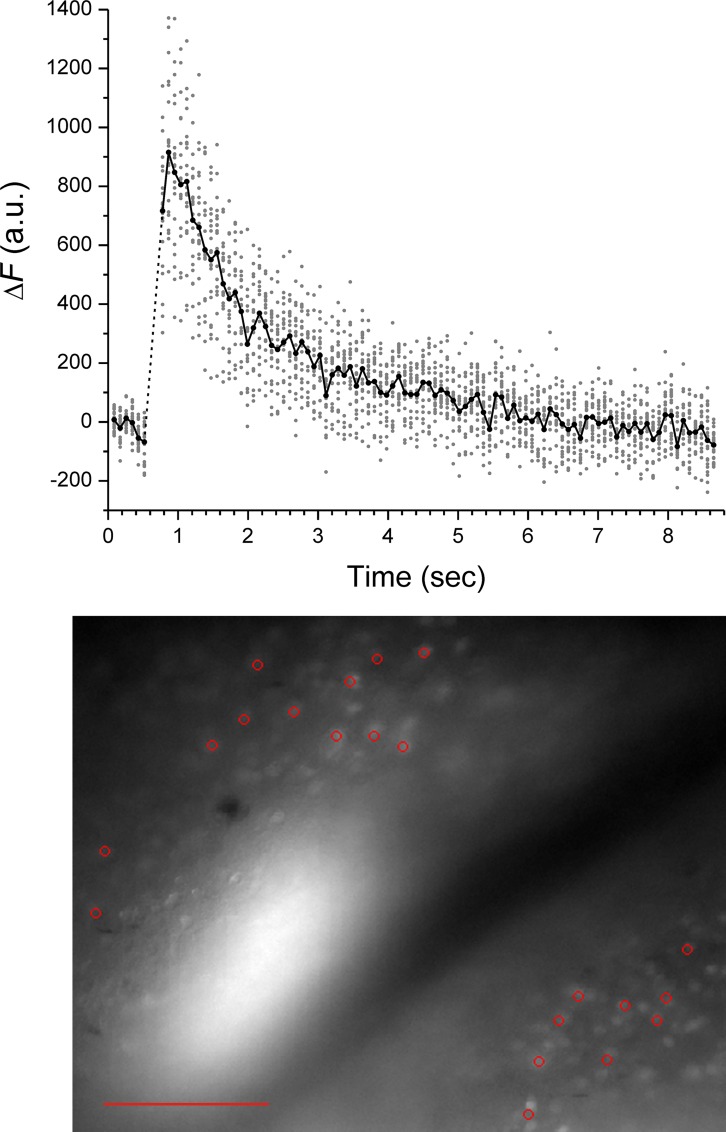
Response of neurons in cortical brain slice to photorelease of *N*-Ncm-Glu. Neurons were loaded with the fluorescent Ca^2+^ indicator, OGB-1, for monitoring intracellular Ca^2+^ signals evoked by glutamate photorelease. In the fluorescence image, red circles mark 21 cells whose responses were analyzed; scale bar represents 100 μm. The center bright area is due to the optical fiber illumination. In the graph, change in OBG-1 fluorescence (Δ*F*) is plotted as function of time. Individual data are shown as gray points; the solid line is the average response of the 21 cells. A 50-ms laser pulse was used for photolysis. The dotted line indicates the interval during which the photolytic laser pulse precluded fluorescence data acquisition.

The use of *N*-Ncm-Glu was also tested in mouse brain slices. Neurons in acutely prepared slices from the auditory cortex were loaded with the fluorescent Ca^2+^ indicator, Oregon Green 488 BAPTA-1 (OGB-1) through incubation with the corresponding acetoxymethyl ester. The inward ionic current activated by glutamate depolarizes the neuronal membrane potential, which triggers Ca^2+^ influx through voltage-gated Ca^2+^ channels. Furthermore, Ca^2+^ influx through Ca^2+^-permeable ionotropic glutamate receptors and intracellular Ca^2+^ signals triggered by metabotropic glutamate receptor activation could also occur. The consequent rise in intracellular free Ca^2+^ concentration can be monitored by imaging OGB-1 fluorescence. The brain slice was perfused with 1 mM *N*-Ncm-Glu and OGB-1 fluorescence was imaged with a CCD camera. Photolytic light pulses were directed to the imaged region through an optical fiber. Fig 10 shows a fluorescence image of the cortical brain slice; 21 cells whose responses were analyzed are marked. The Ca^2+^ signals from the 21 cells in response to glutamate photorelease are also shown in Fig 10. Glutamate photorelease evoked Ca^2+^ signals in all 21 cells; the signals decayed to baseline in ~5 s.

The Ncm cage has several desirable properties. First, it is remarkably easy to synthesize—two-step preparation from commercially available 7-methylcoumarin. Second, the cage can be attached to substrates (e.g., protected amino acids) under mild reaction conditions with good yields. Third, when used in accordance with chemical common sense, it generates chemically stable caged compounds that do not uncage in the absence of light. Finally, Ncm-caged molecules photorelease with useful quantum efficiency. These characteristics make Ncm a useful cage for biological applications. Indeed, Ncm-caged amino acids have already been used to investigate the mechanisms underlying information processing by the dendrites of neurons in the hippocampus [27, 67–69], the maturation of neural circuits in the developing brain [70, 71], as well as the organization and plasticity of neural networks in the auditory cortex [72–74]. These studies amply illustrate the utility of the Ncm cage. Current efforts are focused on extending the chromophore to enable robust absorption in the visible wavelength range, while maintaining the quantum efficiency of photorelease.

## Supporting Information

S1 Fig HPLC determination of amino acids. (PDF)

S2 Fig Spontaneous hydrolysis of *O*-Ncm-Gly. (PDF)

S3 Fig Spontaneous hydrolysis of γ-*O*-Ncm-Glu. (PDF)

S4 Fig Ncm chromophore ring opening is fully reversible. (PDF)

S1 Text Kinetics of the reaction of 7-formyl-6-nitrosocoumarin with glutathione. (PDF)

S2 Text Effect of inner filtering contributed by the photolysis product. (PDF)

S3 Text Estimating the *pK*_a_ values of the α-amino groups of *N*-Ncm amino acids. (PDF)

S4 Text Comparing the bimolecular rate constants for reaction of glutathione with 2-nitrosobenzaldehyde and with 7-formyl-6-nitrosocoumarin. (PDF)
