# Supplementary figures and images for: Ncm, a Photolabile Group for Preparation of Caged Molecules: Synthesis and Biological Application
Source: PLoS One. 2016 Oct 3;11(10):e0163937. doi: 10.1371/journal.pone.0163937 (PMC5047466; doi:10.1371/journal.pone.0163937)

**S2 Fig. HPLC determination of amino acids**

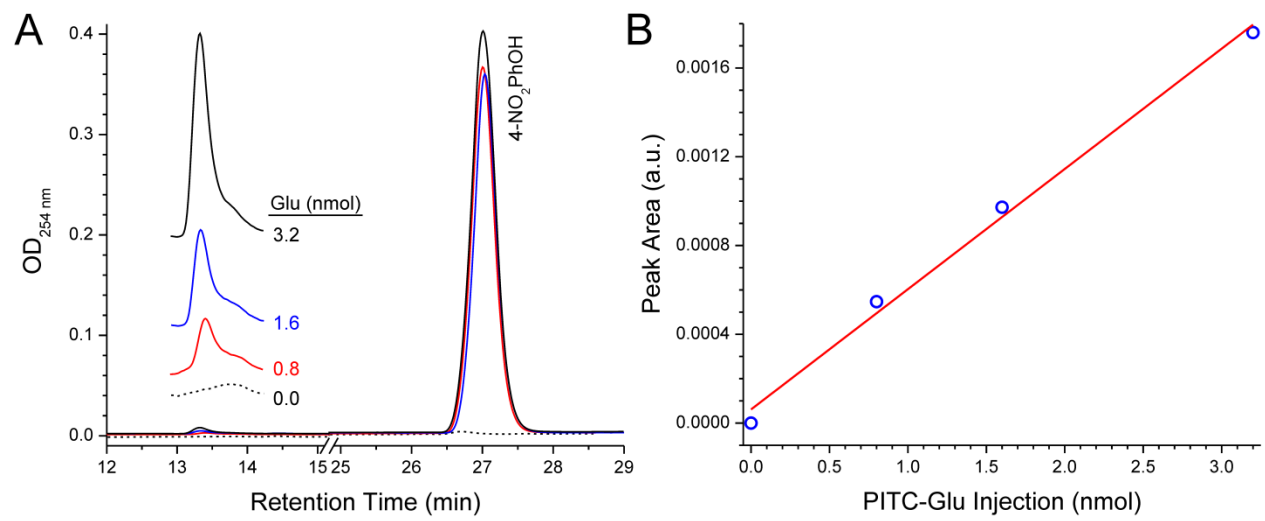

Supplement: S1 Fig — (PDF) [file pone.0163937.s001.pdf]

**S3 Fig. Spontaneous hydrolysis of *O*-Ncm-Gly**

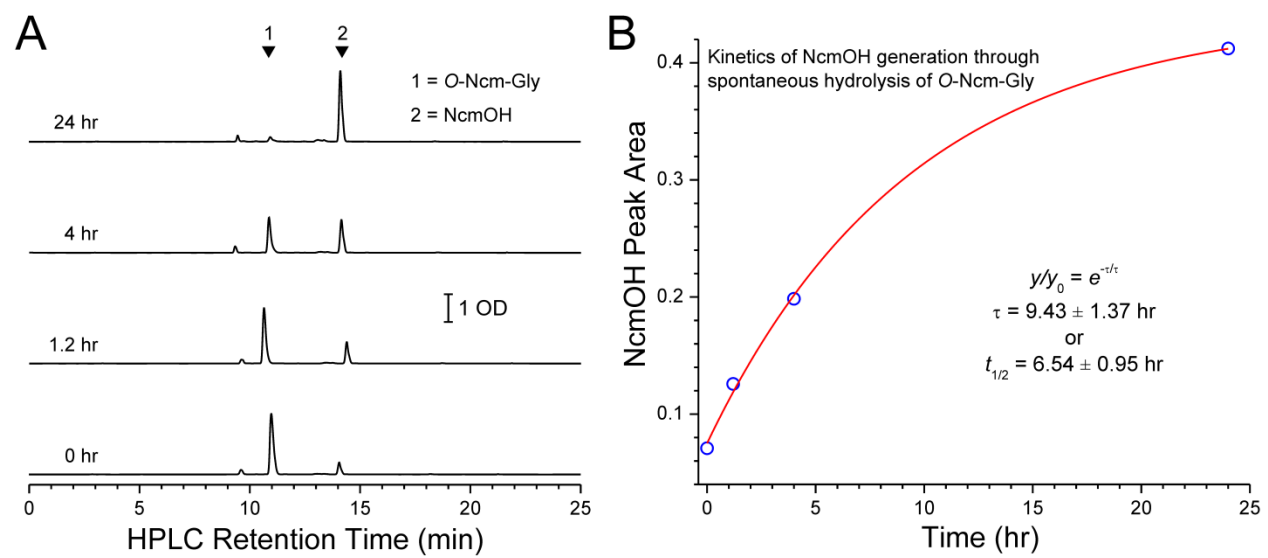

Supplement: S2 Fig — (PDF) [file pone.0163937.s002.pdf]

**S4 Fig. Spontaneous hydrolysis of  $\gamma$ -O-Ncm-Glu**

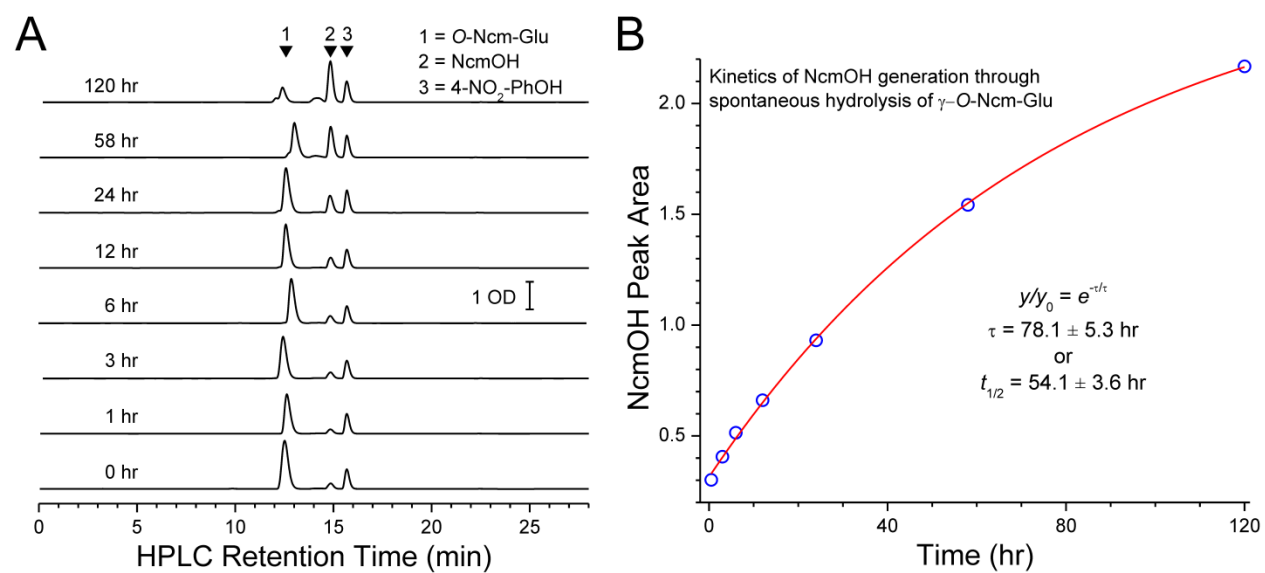

Supplement: S3 Fig — (PDF) [file pone.0163937.s003.pdf]

**S5 Fig. Ncm chromophore ring opening is fully reversible**

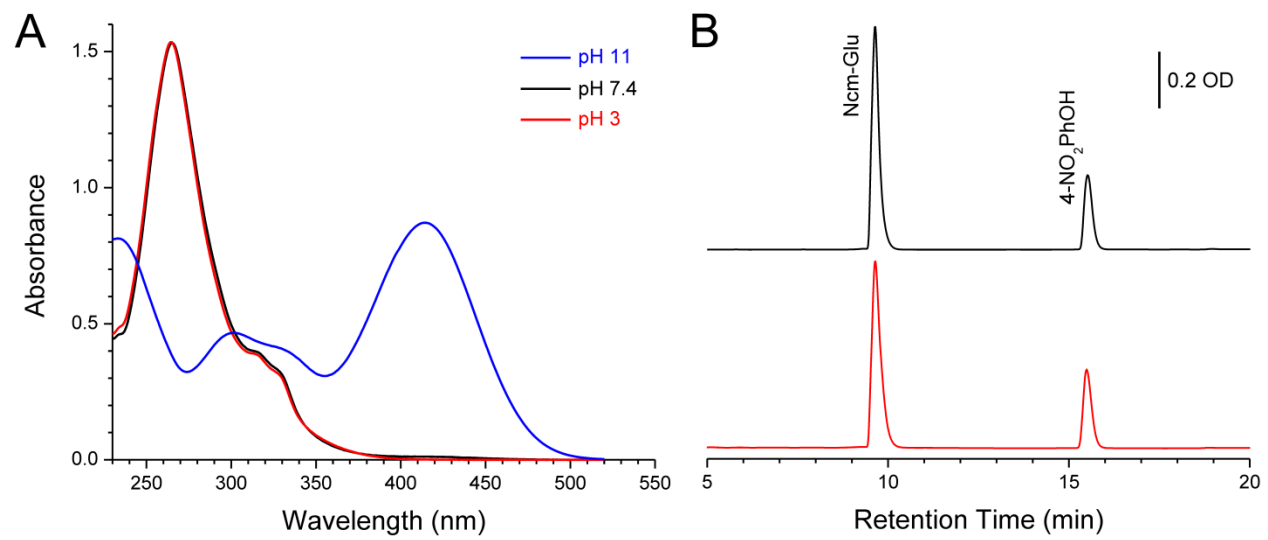

Supplement: S4 Fig — (PDF) [file pone.0163937.s004.pdf]
