# Supplementary material for: Ncm, a Photolabile Group for Preparation of Caged Molecules: Synthesis and Biological Application
Source: PLoS One. 2016 Oct 3;11(10):e0163937. doi: 10.1371/journal.pone.0163937 (PMC5047466; doi:10.1371/journal.pone.0163937)
Supplement: S1 Text — (PDF) [file pone.0163937.s005.pdf]

S1 Text. Kinetics of the reaction of 7-formyl-6-nitrosocoumarin with glutathione

Reaction of a thiol with a nitrosoarene proceeds with thiol addition to the nitroso group [1], which disrupts the nitroso chromophore and thus can be monitored spectrophotometrically. We ran the reaction of 7-formyl-6-nitrosocoumarin (25 μM) with glutathione (GSH, 50 μM) in 10 mM sodium phosphate buffer (pH 7.0) at 24 °C, and monitored the absorbance change at 328 nm. Measurements were in triplicate. The reaction may be represented as

$$\text{Ar-N=O} + \text{GSH} \rightarrow \text{Ar-N(OH)SG}$$

The integrated rate equation, expressed in terms of the biomolecular rate constant, k, the starting concentrations of the reagents, $[\text{ArNO}]_0$ and $[\text{GSH}]_0$, and the extent of reaction, x, is

$$kt = \frac{1}{[\text{GSH}]_0 - [\text{ArNO}]_0} \ln \frac{[\text{ArNO}]_0([\text{GSH}]_0 - x)}{[\text{GSH}]_0([\text{ArNO}]_0 - x)}$$

Making use of the initial condition that $[\text{GSH}]_0 = 2[\text{ArNO}]_0 = 5 \times 10^{-5}$ M and solving for x yields

$$x = [\text{GSH}]_0 \left[1 + \frac{1}{e^{-\,0.5[\text{GSH}]_0\,kt} - 2}\right]$$

Therefore, the following function was fit to the kinetic data:

$$y = y_0 + S\left[1 + \frac{1}{e^{-k_{\text{app}}t} - 2}\right]$$

where $k_{\text{app}} = 0.5[\text{GSH}]_0 k$, S is a scaling factor that incorporates $[\text{GSH}]_0$ and relates concentration change to absorbance change, and y_0 accounts for any minor shifts in baseline. A fit to one of the data set is shown as Fig. 8C in the main text, and reproduced below.

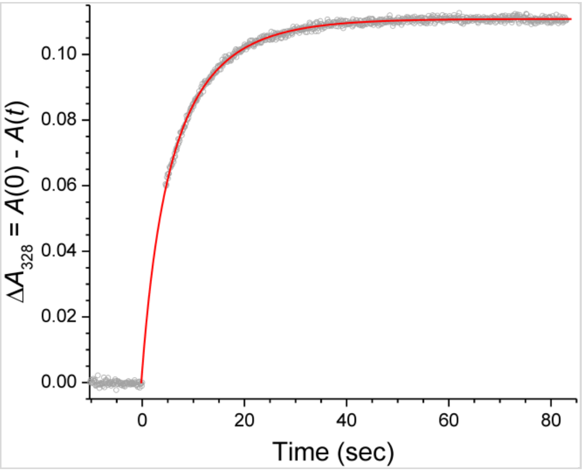

The data are well described by the fitting function, with adjusted $R^2 = 0.99588 \pm 0.0001819$ (n = 3). Averaging values from fits to three data sets, the bimolecular rate constant is $k = (3.86 \pm 0.10) \times 10^3$ $M^{-1}s^{-1}$. As expected, the baseline parameter is small, being $x_0 = 0.004 \pm 0.005$ (n = 3).

1. Klehr H, Eyer P, Schäfer W. On the mechanism of reactions of nitrosoarenes with thiols. Formation of a common intermediate "semimercaptal". *Biol Chem Hoppe-Seyler*. 1985; 366(2):755 – 760. doi: 10.1515/bchm3.1985.366.2.755.
