# Supplementary material for: Ncm, a Photolabile Group for Preparation of Caged Molecules: Synthesis and Biological Application
Source: PLoS One. 2016 Oct 3;11(10):e0163937. doi: 10.1371/journal.pone.0163937 (PMC5047466; doi:10.1371/journal.pone.0163937)
Supplement: S2 Text — (PDF) [file pone.0163937.s006.pdf]

S6 Text. Effect of inner filtering contributed by the photolysis product

As shown in Fig. 7A in the main text (reproduced below), photolysis of the *N*-Ncm amino acids generates photoproducts that absorb more strongly at long wavelengths compared to the starting compounds. Therefore, the photoproducts constitute an inner filter that can absorb,

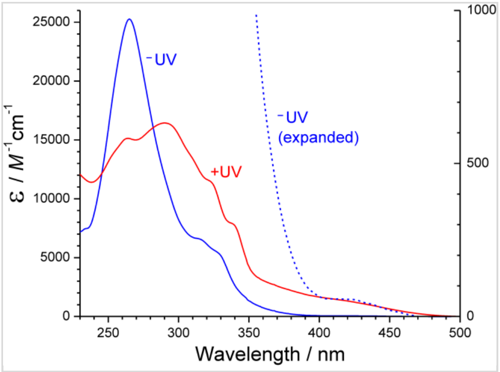

and thus attenuate, the photolysis light beam. To estimate the inner filter effect quantitatively, we need to know the absorbance difference between the photoproduct and the starting compound at the wavelength of photolysis (355 nm). At 355 nm, the photoproducts absorb more than the starting Ncm compound by a factor of 3.44. For the computation, we define the following terms:

f, the factor by which the photoproduct (P) absorbs more than the starting compound (SC);

ε, the extinction coefficient of the starting compound;

C_0, the initial concentration of the starting compound;

α, the extent of photoconversion; $0 \leq \alpha \leq 1$;

τ, the exponential lifetime of the photoreaction (related to the half-life by $t_{1/2} = \ln 2 \cdot \tau$);

t_α, the time required to reach photoconversion fraction α; $t_\alpha = -\ln(1-\alpha)\cdot\tau$.

The total absorption by SC (A_{SC}) up to α fractional photoconversion is represented by

$$A_{SC} = \epsilon C_0 \int_0^{t_\alpha} e^{-\frac{t}{\tau}} dt = \epsilon C_0 \left[-\tau e^{-\frac{t}{\tau}}\right]_0^{t_\alpha} = -\epsilon C_0 \tau \left[e^{\frac{-[-\ln(1-\alpha)]\cdot\tau}{\tau}} - 1\right] = \epsilon C_0 \tau \alpha$$

The total absorption by P (A_P) up to α fractional photoconversion is represented by

$$A_P = f\epsilon C_0 \int_0^{t_\alpha} \left(1 - e^{-\frac{t}{\tau}}\right) dt = f\epsilon C_0 \left[t + \tau e^{-\frac{t}{\tau}}\right]_0^{t_\alpha} = f\epsilon C_0 \left[t_\alpha + \tau e^{-\frac{t_\alpha}{\tau}} - \tau\right] = -f\epsilon C_0 \tau[\alpha + \ln(1-\alpha)]$$

At any fractional conversion, α, the fraction of total absorption due to the starting compound is given by

$$F_{SC} = \frac{A_{SC}}{A_{SC} + A_P} = \frac{\alpha}{\alpha - f[\alpha + \ln(1-\alpha)]}$$

This expression gives finite values for $0 < \alpha < 1$, but is indeterminate at the extrema, α = 0 and α = 1. At the extrema, application of L'Hôpital's Rule gives the correct F_{SC} values of 1 and 0, respectively.

Our quantum yield determinations were performed at 10% photoconversion (α = 0.1), which gives $F_{SC} = 0.844$. Therefore, to account for the inner-filter effect, the apparent quantum yield should be adjusted by F_{SC} to give the true quantum yield, $\Phi = \Phi_{app}/F_{SC} = \Phi_{app}/0.844$.
