# Supplementary material for: Ncm, a Photolabile Group for Preparation of Caged Molecules: Synthesis and Biological Application
Source: PLoS One. 2016 Oct 3;11(10):e0163937. doi: 10.1371/journal.pone.0163937 (PMC5047466; doi:10.1371/journal.pone.0163937)
Supplement: S3 Text — (PDF) [file pone.0163937.s007.pdf]

S7 Text. Estimating the *p*K_a values of the α-amino groups of *N*-Ncm amino acids

The *p*K_as of the α-amino groups of the *N*-Ncm amino acids can be estimated by using linear free energy relationships (LFER) [2]. The approach is summarized in the figure below.

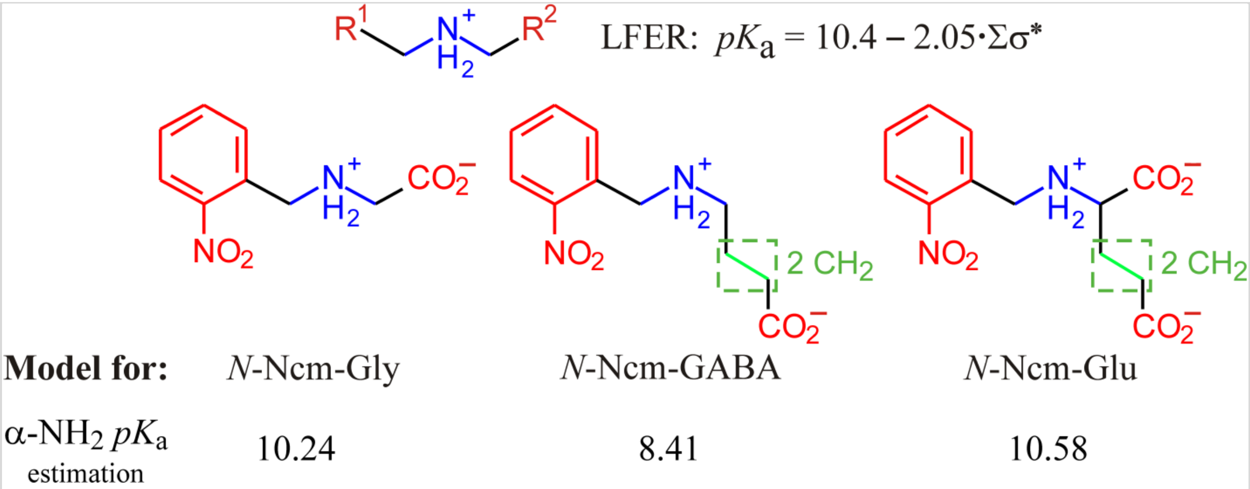

The Taft equation (LFER) for estimating the *p*K_a of a secondary amine with the general formula $R^1CH_2NHCH_2R^2$ is *p*K_a = 10.4 – 2.05·Σσ* [1], where σ* is a substituent constant, and Σσ* represents the summation of all σ* values applicable to a particular molecule. Generally, each additional CH_2 between a functional group and the acidic or basic center attenuates the σ* of the functional group by a factor of 0.4. Finally, σ* is not available for the 6-nitro-7-coumarinyl group; therefore, we use the σ* for 2-nitrophenyl, which should accurately reflect the effect of a 2-nitroaryl group. Estimation of the amino *p*K_a for the three *N*-Ncm amino acids is summarized below; the Taft equation and σ* values used are tabulated in Perrin et al. [1].

<u>*N*-Ncm-Gly</u>

2-NO_2Ph σ* = +1.14

CO_2^- σ* = –1.06

pK_a = 10.4 – 2.05·[1.14 – 1.06] = 10.236

<u>*N*-Ncm-GABA</u>

2-NO_2Ph σ* = +1.14

γ-CO_2^- σ* = –1.06, attenuated by $(0.4)^2$ due separation by 2 CH_2 groups

pK_a = 10.5 – 2.05·[1.14 – 1.06$(0.4)^2$] = 8.411

<u>*N*-Ncm-Glu</u>

2-NO_2Ph σ* = +1.14

α-CO_2^- σ* = –1.06

γ-CO_2^- σ* = –1.06, attenuated by $(0.4)^2$ due separation by 2 CH_2 groups

pK_a = 10.5 – 2.05·[1.14 – 1.06 – 1.06$(0.4)^2$] =10.584

1. Perrin DD, Dempsey B, Serjeant EP. *pKa Prediction for Organic Acids and Bases*: Springer Netherlands; 1981.
