# Supplementary material for: Ncm, a Photolabile Group for Preparation of Caged Molecules: Synthesis and Biological Application
Source: PLoS One. 2016 Oct 3;11(10):e0163937. doi: 10.1371/journal.pone.0163937 (PMC5047466; doi:10.1371/journal.pone.0163937)
Supplement: S4 Text — (PDF) [file pone.0163937.s008.pdf]

S8 Text. Comparing the bimolecular rate constants for reaction of glutathione with 2-nitrosobenzaldehyde and with 7-formyl-6-nitrosocoumarin

For the reaction of glutathione with 7-formyl-6-nitrososcoumarin, we determined a bimolecular rate constant of $k = (3.86 \pm 0.10) \times 10^3$ $M^{-1}s^{-1}$ (at pH 7.0 and 24 °C). This may be compared with the rate constant for the analogous reaction of GSH with 2-nitrosobenzaldehyde to assess relative reactivity. The procedure for the comparison is described below.

The kinetics of the reaction of glutathione (GSH) with substituted nitrosobenzenes have been studied spectrophotometrically [1,2]. The bimolecular rate constants were found to obey a Hammett-type linear free energy relationship (LFER) [1,2], and at 25 °C, the LFER is

$$\log \frac{k}{k_0} = 1.9\sigma$$

where σ is the substituent constant, k is a bimolecular rate constant, and k_0 is the rate constant for the reference compound nitrosobenzene (i.e., where the substituent is H; $k_0 = 5.69 \times 10^3$ $M^{-1}s^{-1}$ at pH 7.49 at 25 °C) [2]. Although the specific reaction of 2-nitrosobenzaldehyde with GSH was not part of the original data set, one can use the LFER to estimate the rate constant—provided the substituent constant (σ_o) for the formyl substituent is known. Owing to the complexity of interactions with *ortho* substituents [3], there is no comprehensive set of σ_o values that apply consistently to different reaction types (in contrast to σ_p and σ_m). Where σ_o has been determined for the formyl group, the value ranges from 0.75 to 1.02 [4-8]. In combination with the above LFER, this range of σ_o implies a range of estimated k for the reaction of 2-nitrosobenzaldehyde with GSH: 1.51 – 4.93×10^5 $M^{-1}s^{-1}$ (at pH 7.49 and 25 °C).

To compare the two apparent, or "conditional", rate constants (for 7-formyl-6-nitrosocoumarin and 2-nitrosoformaldehyde), one must account for the effect of any pH difference, because the concentration of the nucleophilic thiolate species depends on pH. Knowing the microscopic dissociation constants for GSH, calculating the fraction of total GSH that exists in the thiolate form (f_{GS-}) is straightforward, though tedious [9]. The values for f_{GS-} are 0.0116 and 0.0351, at pH 7.0 and 7.49, respectively—a difference of a factor of 3.02. Thus, k for the reaction of 7-formyl-6-nitrosocoumarin with GSH is expected to be 3.02-fold larger at pH 7.49, or 1.17×10^4 $M^{-1}s^{-1}$. Comparing this with the range of k for 2-nitrosobenzaldehyde, 1.51 – 4.93×10^5 $M^{-1}s^{-1}$, shows that 7-formyl-6-nitrosocoumarin is less reactive towards GSH than 2-nitrosobenzaldehyde by a factor 13 to 42.

1. Diepold C, Eyer P, Kampffmeyer H, Reinhardt K. Reactions of aromatic nitroso compounds with thiols. *Advances in Experimental Medicine and Biology*. 1982; 136B:1173-1181. PubMed PMID: 7344505.
2. Kazanis S, McClelland RA. Electrophilic intermediate in the reaction of glutathione and nitroso arenes. *J Am Chem Soc*. 1992; 114(8):3052-3059. doi: 10.1021/ja00034a043.
3. Charton M. The quantitative treatment of the ortho effect. *Progress in Physical Organic Chemistry*. New York: John Wiley & Sons, Inc.; 1971. p. 235-317.

4. Barlin GB, Perrin DD. Prediction of the strengths of organic acids. *Q Rev Chem Soc*. 1966; 20(1):75-101. doi: 10.1039/QR9662000075.
5. Clark J, Perrin DD. Prediction of the strengths of organic bases. *Q Rev Chem Soc*. 1964; 18(3):295-320. doi: 10.1039/QR9641800295.
6. Pearce PJ, Simkins RJJ. Acid strengths of some substituted picric acids. *Can J Chem*. 1968; 46(2):241-248. doi: 10.1139/v68-038.
7. Dietrich MW, Nash JS, Keller RE. Determination of components in phenol mixtures by nuclear magnetic resonance. *Anal Chem*. 1966; 38(11):1479-1484. doi: 10.1021/ac60243a007.
8. Tribble MT, Traynham JG. Nuclear magnetic resonance studies of ortho-substituted phenols in dimethyl sulfoxide solutions. Electronic effects of ortho substituents. *J Am Chem Soc*. 1969; 91(2):379-388. doi: 10.1021/ja01030a029.
9. Legenzov EA, Sims SJ, Dirda ND, Rosen GM, Kao JP. Disulfide-linked dinitroxides for monitoring cellular thiol redox status through electron paramagnetic resonance spectroscopy. *Biochemistry*. 2015; 54(47):6973-6982. doi: 10.1021/acs.biochem.5b00531. PubMed PMID: 26523485.
